# Genome organization and characteristics of soybean microRNAs

**DOI:** 10.1186/1471-2164-13-169

**Published:** 2012-05-04

**Authors:** Marie Turner, Oliver Yu, Senthil Subramanian

**Affiliations:** 1 Plant Science Department, South Dakota State University, Brookings, SD 57007, USA; 2 Donald Danforth Plant Science Center, St Louis, MO, 63132, USA

**Keywords:** microRNA, Soybean, Genome organization, Evolution, Nodulation

## Abstract

**Background:**

microRNAs (miRNAs) are key regulators of gene expression and play important roles in many aspects of plant biology. The role(s) of miRNAs in nitrogen-fixing root nodules of leguminous plants such as soybean is not well understood. We examined a library of small RNAs from *Bradyrhizobium japonicum*-inoculated soybean roots and identified novel miRNAs. In order to enhance our understanding of miRNA evolution, diversification and function, we classified all known soybean miRNAs based on their phylogenetic conservation (conserved, legume- and soybean-specific miRNAs) and examined their genome organization, family characteristics and target diversity. We predicted targets of these miRNAs and experimentally validated several of them. We also examined organ-specific expression of selected miRNAs and their targets.

**Results:**

We identified 120 previously unknown miRNA genes from soybean including 5 novel miRNA families. In the soybean genome, genes encoding miRNAs are primarily intergenic and a small percentage were intragenic or less than 1000 bp from a protein-coding gene, suggesting potential co-regulation between the miRNA and its parent gene. Difference in number and orientation of tandemly duplicated miRNA genes between orthologous genomic loci indicated continuous evolution and diversification. Conserved miRNA families are often larger in size and produce less diverse mature miRNAs than legume- and soybean-specific families. In addition, the majority of conserved and legume-specific miRNA families produce 21 nt long mature miRNAs with distinct nucleotide distribution and regulate a more conserved set of target mRNAs compared to soybean-specific families. A set of nodule-specific target mRNAs and their cognate regulatory miRNAs had inverse expression between root and nodule tissues suggesting that spatial restriction of target gene transcripts by miRNAs might govern nodule-specific gene expression in soybean.

**Conclusions:**

Genome organization of soybean miRNAs suggests that they are actively evolving. Distinct family characteristics of soybean miRNAs suggest continuous diversification of function. Inverse organ-specific expression between selected miRNAs and their targets in the roots and nodules, suggested a potential role for these miRNAs in regulating nodule development.

## Background

microRNAs (miRNAs) are a class of small RNAs that regulate gene expression primarily in a post transcriptional manner [1-3]. Genes encoding miRNAs are transcribed by RNA polymerase II, and the transcript may be capped and polyadenylated, and may even contain introns [1,4]. In plants, primary miRNA (pri-miRNA) transcripts are processed into mature and active 21–24 nt forms in the nucleus by Dicer-Like (DCL) enzymes in a two-step process. First, pri-miRNAs are cleaved into miRNA precursors (pre-miRNAs) that typically have a hairpin-like structure. Then, the pre-miRNA is cleaved to give rise to the miRNA/miRNA* duplex, a highly complementary short double-stranded RNA molecule with characteristic 2 nt 3’ overhangs. miRNA/miRNA* duplexes are methylated on the 2’ OH group of the last nucleotide (3’ end) by HEN1 and are translocated to the cytoplasm. Methylation is thought to protect miRNA-duplexes from degradation [1,5]. In the cytoplasm, miRNA* is generally degraded and the mature miRNA molecule is incorporated into a RNA-induced silencing complex (RISC), composed of different proteins including the catalytic protein ARGONAUTE (AGO). This complex either directs the cleavage of complementary target mRNAs [2,3,6] or inhibits their translation [7] primarily depending on the extent of sequence complementarity between the miRNA and the target mRNA. The majority of conserved miRNAs regulate transcription factors and signaling elements, although a number of other classes of target genes are being discovered [1,8-11]. miRNAs play crucial roles in many aspects of plant development including organ morphogenesis or patterning primarily by regulating hormone signaling [3,7]. They also play a role in response to abiotic stresses [12-14] and resistance against pathogenic organisms [15].

The availability of complete genome sequence for a number of plant species has allowed a better understanding of miRNA evolution and genome organization. Two mutually non-exclusive models have been proposed for miRNA origin; miRNA genes arise from inverted duplication of protein-coding genes (that eventually become target of the miRNA) or are born randomly from the numerous inverted repeats in the genome [4,7]. Resulting miRNA/target pairs would then be selected through evolution. Evolutionary forces such as duplication, inversion, mutation, amplification, and other types of genetic drift that shape the genome might be the primary events in the origin and evolution of miRNA genes. Identification of numerous young miRNA genes with low expression and few if any targets supports the hypothesis of a rapid birth-death model [16]. The presence of deeply conserved and species-specific miRNAs, in various plant species, points towards a continuous, rapid and uneven evolutionary process of miRNA genes [4,7]. miRNA genes are grouped into families, based on paralogous family members producing identical or nearly identical mature sequences [17].

We previously identified a set of miRNAs from soybean (*Glycine max*) by sequencing two libraries of small RNAs, one from *Bradyrhizobium japonicum*-inoculated and the other from mock-inoculated roots [18]. Subsequently, a number of miRNAs have been identified in soybean [19-24] and other legumes such as *M. truncatula*[25-27], *Phaseolus vulgaris*[12,28] and *Arachis hypogaea*[29]. The identification of nodulation-regulated and legume-specific miRNAs has suggested important roles for these molecules in nodule development [21,25,26,30]. Indeed, the crucial role of miRNAs in nodule development has been demonstrated in both *M. truncatula*[31,32] and soybean [33].

The complete genome sequence of soybean was recently released. It is a 1.1 gigabase genome with approximately 46,430 protein coding genes. Two whole genome duplication events, suggested to have occurred at approximately 59 and 13 million years ago, resulted in a highly duplicated genome with nearly 75% of the genes present in multiple copies [34-36]. In the present study, we identified a number of novel miRNAs and additional family members for known miRNAs. In addition, we compiled all miRNA sequences available in soybean and performed a comprehensive analysis of miRNA distribution in the genome as well as their family organization, mature sequence diversity and target prediction. Expression analysis of selected miRNAs and targets in different soybean organs revealed regulation of organ-specific target gene expression by miRNAs. Overall, our results have provided novel insights on miRNA evolution, diversification and regulation in soybean.

## Results

We previously identified a number of soybean miRNAs through high throughput sequencing of a small RNA library and examining WGS and EST sequences for potential precursors [18]. We speculated that when the complete genome sequence of soybean is available, additional novel miRNAs might be discovered. It was indeed the case and here, we report the identification of a number of novel miRNAs and previously unknown family members for conserved miRNAs in the recently released soybean genome sequence [36].

### Identification of novel miRNAs families and family members in soybean

A primary characteristic of miRNAs that distinguishes them from other small RNAs is that they arise from hairpin forming precursors [1-3,17,37]. We mapped unique sequence reads in our small RNA library to the soybean genome sequence (http://www.phytozome.net; Glyma1.0 [36]) and identified hairpin-forming precursors that give rise to mature miRNAs. Among the 2696 potential hairpins encompassing library reads, 101 (previously unknown) hairpin structures that passed the criteria for annotation of plant miRNAs described by Meyers *et al*. [17] were annotated as miRNAs (see Materials and methods and Table S1 (Additional file 1: Table S1, Sheet1) for details). Details on different primary and secondary criteria for these 101 hairpin structures are presented in Table S1 (Additional file 1: Table S1, Sheet1). In parallel, we performed a BLAST search on the soybean genome sequence using known plant miRNA sequences from miRbase as query to identify additional conserved miRNA sequences. We then analyzed potential hairpin sequences using the above criteria [17] and identified an additional 19 miRNAs. In total, we identified 120 previously unknown hairpin-forming precursors (Table 1 and Additional file 1: Table S1, Sheet1). We then compared the miRNA precursor sequences against miRBase (V17, April 2011) and clustered them into different families. A total of 31 families of miRNAs were identified: 20 families from the library, 7 families from the BLAST search and 4 families from both library and BLAST search (Table 1 and Additional file 1: Table S1, Sheet1). Among these, 8 were novel for soybean: 3 were present in miRbase but had not been identified in soybean and the other 5 were novel families not described before (Additional file 2: Figure S1). The remaining 23 families have previously been identified in soybean [18-21,38]. Thirty six of the previously unknown miRNA loci we identified in the soybean genome (Table 1), were independently reported by other groups [22-24] during the preparation of this manuscript.

**Table 1:** Summary of soybean miRNAs identified in this study

**Soybean miRNA family ID**		**Number of family members**		
		**Previously identified**^**1**^	**Identified in this study**	**Total**
	156	10	12	22
	159	5	4	9
	160	1	5	6
	162	1	2	3
	164	1	11	12
	166	5	14	19
	167	7	1	8
	168	1	1	2
	169	10	4	14
	171	5	12	17
	172	5	5	10
	319	4	7	11
	390	2	5	7
	393	1	10	11
	394	2	1	3
	395	4	5	9
	396	4	2	6
	408	2	1	3
	*530*^*2*^	*0*	*3*	*3*
	*828*	*0*	*1*	*1*
	1512	2	1	3
	1513	1	2	3
	1515	1	1	2
	1516	1	2	3
	*2118/2218*^*2*^	*0*	*2*	*2*
	4416	1	1	2
**gma-new-miR11602**^**2**^		0	1	1
**gma-new-miR21193**		0	1	1
**gma-new-miR13587**		0	1	1
**gma-new-miR14018**		0	1	1
**gma-new-miR50841**		0	1	1
**Total**	**31**	**76**	**120**	**196**

miRNA families that were not described previously in soybean are highlighted in grey. Five novel miRNAs (See also Additional file 2: Figure S1) that have not been described previously in any organism are shown in bold face.^1^ Previously identified and listed in Zhang *et al.* (2008), PMRD [38], Kulcheski *et al.*[24], Song *et al.*[23] or in miRbase (v17).^2^Independently identified by Kulcheski *et al.*[24] or Song *et al.*[23] when the manuscript was under preparation.

### Soybean miRNA genes are primarily intergenic

**Figure 1:**
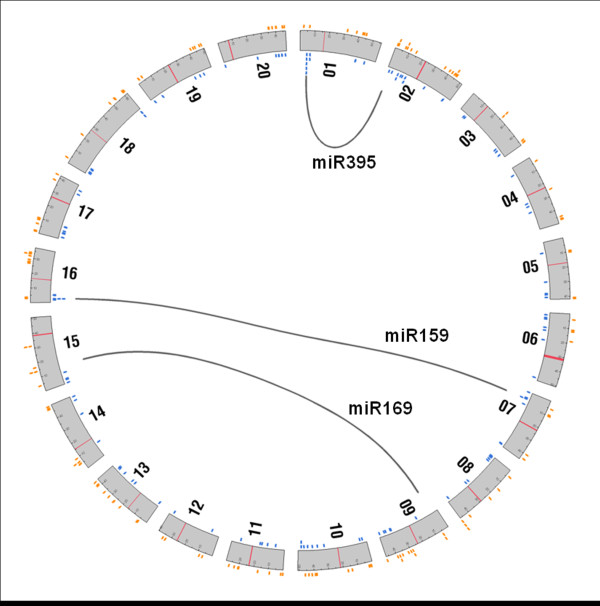
**Genome organization of soybean miRNAs; physical location and tandem duplications. **Each grey box represents a chromosome and the centromere region is indicated by a red band. miRNAs in top strand are shown in orange and the ones in the bottom strand are shown in blue. Grey lines indicate regions with tandemly duplicated miRNA genes and their corresponding synonymous multiplicons.

Next, we examined the organization of all miRNA genes available in the soybean genome (http://www.phytozome.net). We compiled a comprehensive list combining miRNAs identified in this study, all miRNA sequences available in miRbase (which includes soybean miRNAs identified by Subramanian *et al*.[18], Joshi *et al*. [19], Wang *et al*. [21] and Zhang *et al.*[20]) and plant miRNA database [38] and publicly available sequences from articles published during the preparation of this manuscript [22-24]. All these miRNA precursors were examined using miRNA annotation criteria [17] and only those that passed were used for subsequent analyses. There were a number of miRNAs that produced 24nt mature species that passed these criteria. Due to the ambiguity of these miRNAs being heterochromatic siRNAs (hc-siRNAs;[39] or long miRNAs (lmiRNAs; [40,41]), these were also not included in subsequent analyses for genome organization and family characteristics (See Additional file 3: Table S1: Sheet2 for details). A total of 285 miRNA genes representing 108 families were used in these analyses (including 120 genes identified in this study). We determined their physical location in the genome, position with reference to protein-coding genes, and potential duplication. Genes encoding miRNAs were present in all 20 soybean chromosomes with no apparent preference between the top strand and the bottom strand (Figure 1). However, miRNA genes did appear to be preferentially located away from the centromeric regions similar to protein-coding gene [36]. The majority of miRNA genes were located in intergenic regions. However, 7 miRNA genes were intragenic (in CDS or UTR) and 7 others were situated less than 1000 bp from a protein-coding gene (named parent gene and proximal gene respectively; Additional file 3: Table S2). It is possible that transcription of these miRNAs and their parent or proximal genes are co-regulated. For example, gma-new-miR13587 is 748 bp 3’ to Glyma05g36870 and we observed preferential expression in roots for both the miRNA and the parent gene (see Discussion).

### Diversity of duplicated miRNA genes in paralogous genomic regions indicates continuous evolution

**Figure 2:**
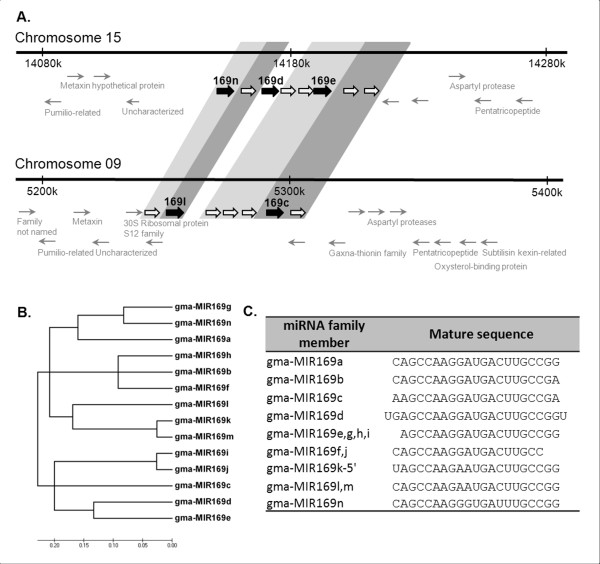
**Tandem duplication of MIR169 genes. A. **Illustration showing a portion of soybean chromosome 15 and its paralogous genomic locus on chromosome 9 with tandemly duplicated MIR169 genes (filled arrows), MIR169-like genes (hollow arrows; see text for explanation) and protein-coding genes (grey arrows). miRNAs and miRNA-like genes with high sequence identity are shown encompassed in background boxes. Annotations specified where available (Arrows not to scale of gene length). **B.** Phylogenetic tree obtained by aligning the precursor sequences of all soybean MIR169 genes. **C.** List of all mature sequences identified for miR169 family (See Table S1 for updated miRbase IDs for miR169 family members).

We then examined clustering of miRNA genes in the genome as this might be evidence for continuous evolution and diversification of function [16,42]. We identified four families of miRNAs (miR159, miR169, miR395 and gma-miR-Seq14) that had at least one locus with tandemly duplicated genes. The soybean genome is suggested to have undergone two different genome duplications: the first ~59 mya and the most recent ~13 mya [34-36]. To determine if miRNA duplication occurred more recently, we compared a 100 kb region surrounding the miRNA gene with its duplicated paralogous region in the soybean genome (Figure 1). Genes encoding MIR169n, -d and -e are tandemly duplicated on chromosome 9. The paralogous region on chromosome 15 has two miRNA genes, MIR169l and -c (Figure 2). However, both regions contain a number of ‘MIR169-like’ genes from which either no mature miRNA has been detected or the ones detected did not fit the criteria established by Meyers *et al.*[17]. The organization of MIR169 and MIR169-like genes in these regions (Figure 2) indicates that both regions might have had same number of MIR169-like genes and possible “birth” (e.g. miR169d) and “death” of genes (e.g. MIR169-like genes) occurred more recently, resulting in a diverse set of family members. Indeed, the absence of a MIR169d-like gene in one of the paralogous genomic loci (Figure 2) and high sequence similarity between MIR169d and -e (Phylogenetic tree, Figure 2B) suggested that MIR169d might have originated from MIR169e. A number of the paralogous MIR169-like genes had highest similarity to MIR169e, suggesting that they all might have originated form the same precursor. Consistently, miR169e shares the same mature sequence with a number of other family members (Figure 2). Similar observations were made for miR395 and miR159 gene families, suggesting continuous evolution of these families as well. However, unlike MIR169 genes, head to head (MIR159) and tail to tail (MIR395) duplications in addition to tandem duplications were observed in these families (Additional file 2: Figure S2). It should be noted that MIR159, MIR169 and MIR395 genes are tandemly duplicated in other plant species as well (e.g. *M. truncatulaArabidopsis*). In addition to these conserved miRNAs, a soybean specific miRNA family, miR-Seq14, had tandemly duplicated loci on chromosome 9. In conclusion, differences in orientation and number of tandemly duplicated miRNA genes between paralogous genomic regions seem to indicate that soybean miRNAs continue to evolve and diversify in function.

### Conserved miRNA families are larger in size compared to legume- or soybean-specific families

**Figure 3:**
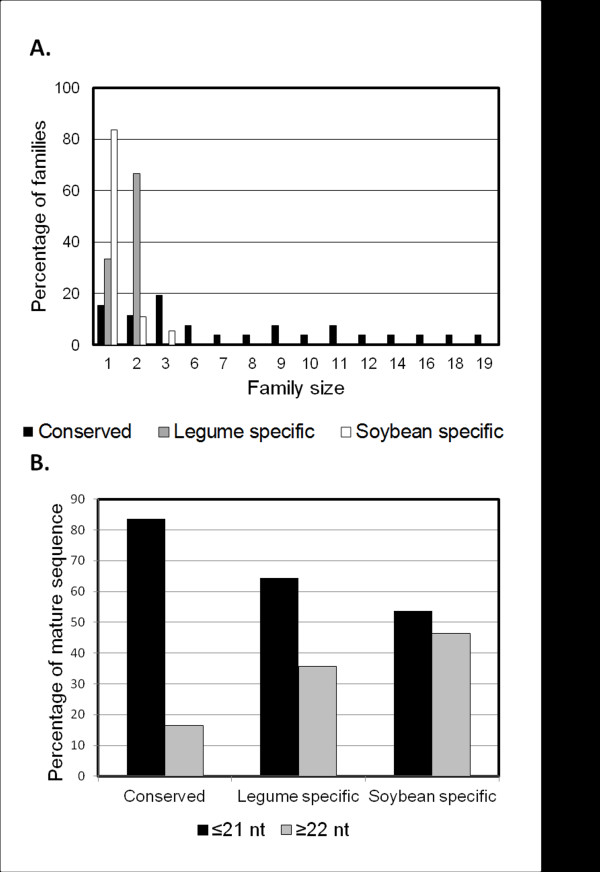
**Soybean miRNA family size and mature sequence length. A. **Size of different classes of soybean miRNA families. Conserved (black bars, 26 families), legume-specific (grey bars, 9 families) and soybean-specific (white bars, 73 families) families had distinct characteristics. Family size was statistically different between conserved and both legume and soybean-specific families (Student’s T-test, P = 10^-6^and 10^-6^respectively), and different between legume and soybean-specific families (Student’s T-test, P = 0.04). **B.** Percentage of loci producing ≤21 nt (black bars) or ≥22 nt (grey bars) long mature miRNAs in each conservation class. Mature sequence length was statistically different between conserved and soybean-specific families (Student’s T-test, P = 0.01) as well as conserved and legume-specific families (Student’s T-test, P = 0.03).

Next, we compared different characteristics of soybean miRNA families. It has been observed in Arabidopsis that a number of characteristics such as family size, number of different mature sequences and the length of mature sequence are distinct between conserved and species-specific miRNA families [16,42,43]. We classified soybean miRNAs into three categories according to the level of phylogenetic conservation, i.e. presence of homologs in other species: 1. Conserved (found in multiple families of plants), 2. Legume-specific (found only in members of *Fabaceae*) and 3. Soybean-specific (i.e. found only in *Glycine max*; when a miRNA was found also in *G. soja*, it was classified as legume-specific) and examined the above family characteristics. In soybean, conserved miRNA families were the most diversified in terms of family size (Figure 3A). They ranged in size from 1 member (e.g. gma-miR828) to 19 members (e.g. miR166) with an average of 7 members per family. In contrast, legume-specific families had just one (1/3 of the families) or two members (2/3 of the families). Similarly, the large majority of soybean-specific families (83.6%) had just one member. Family size was indeed highly statistically different between conserved and both legume and soybean-specific families (Student’s T-test, P = 0.00006 and 0.00002 respectively). Family size was also statistically different between legume and soybean-specific families (Student’s T-test, P = 0.04). In conclusion, conserved miRNA families are larger in size with multiple family members whereas legume-specific and soybean-specific miRNA families are smaller with fewer members in agreement with reports in Arabidopsis.

### The majority of conserved and legume-specific miRNA families produce 21 nt mature sequences and have distinct nucleotide distribution compared to soybean-specific families

The size of the mature sequence is also closely related to the level of conservation of miRNA families in Arabidopsis [42]; most of the conserved families produced 21 nt long mature miRNAs whereas most of the species-specific families produced 22 nt long mature miRNAs. In soybean, the majority of conserved and legume-specific families produce 21 nt long mature miRNAs while about half the number of soybean-specific families produce 22 nt mature miRNAs (Figure 3B). Mature sequence length was statistically different between conserved and soybean-specific families (Student’s T-test, P = 0.01) as well as conserved and legume-specific families (Student’s T-test, P = 0.03). A preference for U in the 5’end of mature miRNAs has been identified in plants [42,44]. In soybean as well, there was a preference for U at the 5’ end of miRNAs (Additional file 2: Figure S3) irrespective of the conservation class. The identity of the first base seemed to be a family-specific characteristic. For example, among the conserved families, miR159, miR390 and miR395 had a preference for A at the 5’ end of mature miRNAs (Additional file 1: Table S1, sheet1). In soybean, a preference for C as the 19^th^ base was previously reported [20]. This was indeed true for conserved families but not for the legume- and soybean-specific families (Additional file 2: Figure S3). In conclusion, mature sequence length and nucleotide preference at the 19^th^ position of mature miRNA sequence was found to be distinct between conserved, legume-specific and soybean-specific families.

### Conserved miRNA families produce less diverse mature sequences compared to legume- or soybean-specific families

The ultimate determinant of the range of targets regulated by a particular miRNA depends on complementarity between mature miRNAs and mRNA targets. We examined the number of different mature miRNAs produced by different miRNA families. In 46% of conserved miRNA families, mature miRNAs produced by different members were identical in sequence. For example, miR164 has 12 family members, but all of them produce identical mature miRNAs (Additional file 3: Table S1, Sheet2). On the other hand, in legume- and soybean-specific miRNA families, mature miRNAs from each family member was often distinct in sequence. For example, 6 legume-specific miRNA families had two family members each and in 5 of these families, each family member produced a distinct mature miRNA. Similarly, in 3 of the 4 soybean-specific miRNA families that had 3 family members, each family member produced a distinct mature miRNA (Additional file 3: Table S1, Sheet2). On average, each mature miRNA sequence was encoded by 2.6, 1.1 and 1.1 members respectively in conserved, legume-specific and soybean-specific miRNA families. Differences in diversity of mature sequence per loci were statistically significant between conserved families and both legume- and soybean-specific families (Student’s T-test, P = 0.002 and 0.002 respectively). There seems to be a correlation between the diversity in mature miRNA sequence and the level of conservation of miRNA families.

### Conserved and legume-specific miRNAs might regulate a large number of genes with similar function whereas soybean-specific miRNAs might regulate fewer genes with diverse functions

Understanding the role of miRNAs in biological processes involves the identification and analysis of their downstream targets. Target prediction is also an indicator of miRNA evolution as conserved miRNAs often regulate similar target genes whereas species-specific miRNAs might have diverse targets or often no predicted targets [16]. We used a custom perlscript to predict targets for soybean miRNAs (Table 2 and Additional file 1: Table S3) according to the criteria previously described [8,9]. It is worth mentioning that among the targets that we have predicted, 90 were confirmed using degradome analysis (Additional file 1: Table S3) [23]. The majority of predicted targets for conserved miRNAs were in agreement with what was previously described in other species such as Arabidopsis. For example, the targets of miR156 family belong to Squamosa Protein binding-Like (SPL) proteins and targets of miR167 to Auxin Response Factors (ARF6 and 8). Most of the legume-specific miRNA families seemed to regulate disease-resistance proteins, consistent with the plant family or species-specific nature of these genes. Soybean-specific miRNA gene families appeared to target fewer genes with a more diverse range of functions. Indeed, for 27 out of 84 soybean-specific families, no targets were predicted suggesting that these might still be evolving. Such an observation is also supported by results from recent degradome analysis-based identification of soybean miRNA targets [23]. Conserved and legume-specific miRNA families appeared to regulate a higher number of targets compared to soybean-specific miRNA families (Table 2). In terms of number of targets predicted, there was highly significant difference between conserved and soybean-specific miRNA families (Student’s T-test, P < 10^-8^). These observations are in agreement with what has been reported as characteristics of conserved and species-specific families in Arabidopsis [16,42,43].

**Table 2:** Average number of targets per mature sequence in conserved, legume-specific and soybean-specific miRNA families

**miRNA families**	**Average number of targets predicted per mature sequence**	**Mature sequences with predicted targets**^**1**^	**Principal predicted function**
Conserved	10.2	72/79	Transcription factors (15/26 families)
Legume-specific	8.8	14/14	Disease resistance proteins (4/9 families)
Soybean-specific	3.4	57/84	Variable

^1^Number of mature sequences for which targets were present, over the total number of mature sequences.

### Dual targeting by legume-specific miRNAs might initiate tasiRNA production

In some cases, miRNAs from two different families appeared to target the same gene at different positions (Additional file 4: Table S3). Often these miRNA pairs appeared to regulate a family of genes. For example, miR2109 and miR1510 were predicted to target the same 5 genes, but at different positions (~570 and 600nt apart) and all of them are annotated as potential disease-resistance genes. This observation suggested potential generation of trans-acting siRNAs (tasiRNAs) from these target genes via the “two-hit model” [9,45,46]. We examined all available small RNA libraries from soybean (NCBI GEO archive) for generation of tasiRNAs (i.e. ‘phased’ reads from both strands) from these target genes. We examined the number of reads in phase (21nt) with the predicted miRNA cleavage site. The number of reads in phase was compared to total number of reads from the same strand. Among all soybean targets predicted to be regulated by two different miRNAs, three (Glyma09g07290, Glyma09g39260 and Glyma16g27790 – all encoding pentatrichopeptide repeat-containing proteins and potential targets of miR1508 and miR4413) produced phased reads whose abundance was above the median number of reads from other positions (data not shown). Perhaps, regulation by multiple miRNAs is a mechanism for fine-tuning gene expression, either as a fail-safe mechanism or to generate tasiRNAs rather than a random coincidence. Indeed, during the preparation of this manuscript, a large scale analysis of small RNAs and degradomes of *M. truncatula* and other legumes identified and validated the production of phased siRNAs from NBS-LRR genes [47] either through initiation by 22nt miRNAs [48] or the two-hit model [9,45,46].

### Validation of predicted targets by RLM-RACE

A subset of targets with high target prediction scores (ratio of minimum free energy compared to perfect complementary pairing between miRNA and target), higher expression in roots and/or nodules (soybean gene atlas; http://digbio.missouri.edu/soybean_atlas/; [49]) and known functional annotation were selected for experimental validation of target cleavage (modified 5’-RACE assay [18]). Among the five targets of miR169c and miR169g selected for analysis, four were confirmed by 5’-RACE analysis; similarly, the two targets of miR2118/2218 assayed were also validated (Table 3; Additional file 4: Table S4).

**Table 3:** Validation of selected miRNA targets by a modified 5’RACE assay

**miRNA**	**Target name and function**	**Target prediction score**^**1**^	**5’RACE result**^**2**^	
**gma-miR169c and gma-miR169g^**3**^**	Glyma10g10240 TRANSCRIPTION FACTOR NF-Y ALPHA-RELATED	0.78	5’ UAGGCAACUCAUCCUUGGCUC 3’| ||||| |||||||||||| **(A/G)GCCGUUCAGUAGGAACCGA(A**	4/6
	Glyma15g18970 TRANSCRIPTION FACTOR NF-Y ALPHA-RELATED	0.79	5’ CAGGCAAAUCAUCCUUGGCUU 3’||||| ||||||||||||| **(A/G)GCCGUUCAGUAGGAACCGA(A)**	11/16
	Glyma17g05920 RANSCRIPTION FACTOR NF-Y ALPHA-RELATED	0.79	5’ CUGGCAAAUCAUCCUUGGCUU 3’||||| ||||||||||||| **(A/G)GCCGUUCAGUAGGAACCGA(A)**	7/15
	Glyma19g38800 TRANSCRIPTION FACTOR NF-Y ALPHA-RELATED	0.72;0.78	5’ UAGGCAAUCCAUCCUUGGCUC 3’| ||||| ||||||||||| **(A/G)GCCGUUCAGUAGGAACCGA(A)**	7/15
**gma-miR2118/2218a**	Glyma12g03040 LRR;TIRdomain; NB-ARCdomain;Apoptotic ATPase	0.7	5’ AUGGAACCGGUGGAAUUGGCAA 3’|||| |||||||| ||||| **AUCCUUACCCACCUUAGCCGUU**	14/16
	Glyma20g06780 LRR;TIRdomain; NB-ARCdomain;Apoptotic ATPase	0.71	5’ AUGGAACUGGUGGAAUUGGCAA 3’|||| |||||||| ||||| **AUCCUUACCCACCUUAGCCGUU**	3/13

In conclusion, soybean miRNA genes are primarily intergenic, but a small percentage was also intragenic or close to protein-coding genes suggesting co-regulation of miRNAs and protein-coding genes. Differences in the orientation and number of tandem duplications and clustering of soybean miRNA genes between paralogous loci, indicated recent and continuous evolution and diversification of soybean miRNAs. Family size, diversity, length and nucleotide distribution of mature miRNAs, and the number and range of targets regulated were distinct between conserved, legume-specific and soybean-specific miRNA families. Interestingly, legume-specific families presented characteristic of both conserved and soybean-specific families, depending on the criteria considered, but seem more closely related to soybean-specific families.

### Organ-specific/preferred expression of soybean miRNAs

**Figure 4:**
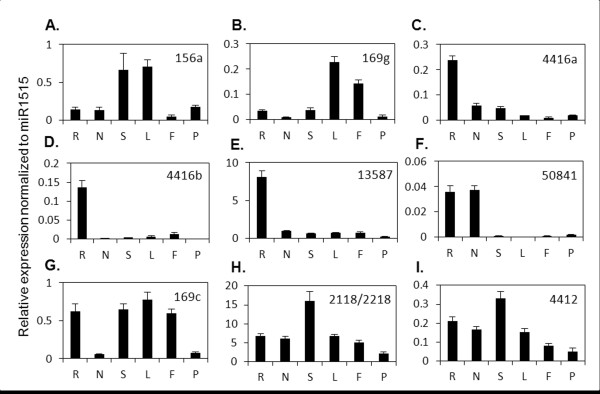
**Expression of selected miRNAs in different soybean organs. A-B. **miRNAs preferentially expressed in aerial organs (miR156a and miR169g). **C-F. **miRNAs preferentially expressed in root organs (miR4416a-b, gma-new-miR13587 and gma-new-miR50841). **G-I. **miRNAs with no clear organ specificity. Data shown are expression levels relative to that of miR1515. Error bars indicate SD between replicates (miR169c, miR2118/2218 and miR4412). Root (R), Nodule (N), Stem (S), Leaf (L), Flower (F) and Pod (P).

As discussed earlier, multiple members of conserved miRNA families often encode near identical mature miRNAs. Therefore, functional diversity among them is likely due to differences in their temporal and spatial expression patterns. We examined the expression of nine selected miRNAs in different soybean organs (Figure 4) by RT-qPCR. miR169 (two different mature forms, 169c and g) was chosen for its previously identified role in nodulation [31]; miR2118/2218 as part of a legume specific family [25]; two new miRNAs identified in this study and four other miRNAs (for which expression data was not available) were randomly selected (see Additional file 2: Figures S4, for dissociation curves and amplification efficiency). Expression levels were normalized to miR1515 which is uniformly expressed in different soybean organs [33].

We observed clearly distinct organ-specific/preferred expression of these miRNAs. For example, miR156a and miR169g appeared to be preferentially expressed in aerial organs (Figure 4A and B) whereas miR4416a, miR4416b, gma-new-miR13587 and gma-new-miR50841 had a preferential expression in root organs (Figure 4C-F). Among the four miRNAs with preferential expression in the root, gma-new-miR50841 was also highly expressed in the nodules. miR169c, miR2118/2218 and miR4412 were expressed in all organs and did not seem to have a clear organ specificity (Figure 4G-I). Among the miRNAs belonging to the same family, both miR4416a and miR4416b had root-preferred expression profiles. In contrast, miR169c and miR169g had clearly distinct organ-specific expression profiles; while miR169c was expressed at high levels in the root, stem, leaves and flowers, miR169g was expressed primarily in leaf and flowers.

In conclusion, different organ specificities in miRNA expression were identified, four miRNAs being specific to root organs including one that was expressed in roots and nodules.

### Potential regulation of organ-specific target gene expression by miRNAs

**Figure 5:**
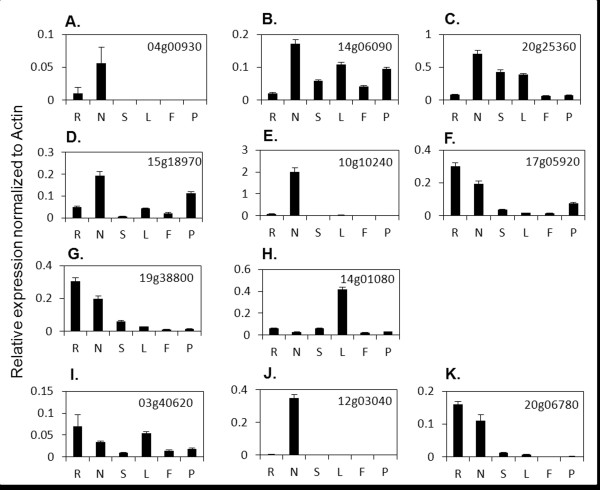
**Expression of selected targets of miRNAs in different soybean organs. **Data shown are expression levels relative to that of Actin. Targets are grouped according to the cognate miRNA: **A-C**. Targets of gma-new-miR13587, **D-G. **Targets of miR169c and –g, **H. **Target of miR169c, **I. **Target of miR156 and **J-K. **Targets of miR2118/2218. Root (R), Nodule (N), Stem (S), Leaf (L), Flower (F) and Pod (P).

We also examined the expression of selected targets of these miRNAs in various soybean organs. Interestingly, we observed an inverse correlation between pairs of miRNAs and targets among different organs. Most targets of aerial organ-specific/preferred miRNAs had a root organ-specific/preferred expression suggesting that organ-specific expression of targets could be regulated by miRNAs. For example, the three potential targets of gma-new-miR13587 had highest expression in the nodules and lower expression in root (Figure 5A-C). On the contrary, gma-new-miR13587 was highly expressed in the roots, but very poorly expressed in the nodules (Figure 4E), suggesting that spatial restriction of miRNA-target pairs might be one of the mechanisms governing nodule-specific gene expression. Similarly, miR169c and miR169g, and one of their targets Glyma15g18970 showed complementary organ-specific expression; while the target gene was highly expressed in nodules and pods (Figure 5D), the miRNA genes were very poorly expressed in these organs (Figure 4B and G). Our results suggest potential regulation of organ-specific gene expression (e.g. in nodules) by spatial distribution of the miRNAs in soybean.

## Discussion

### Identification of novel miRNAs and family members

We previously identified 55 families of soybean miRNAs through high throughput sequencing of a small RNA library and analyzing the data using WGS and EST sequences [18]. Using conserved miRNAs as ‘internal control’ we estimated that a number of miRNA family members were not identified primarily due to non-availability of either genome sequence data or assembly. Indeed, re-examination of our library with the recently released soybean genome assembly (http://www.phytozome.net; Glyma1.0; [36]), allowed us to identify 5 new miRNAs and 109 novel family members for previously known miRNAs. We used the criteria defined to annotate plant miRNAs [17] and our results have significantly enhanced our knowledge on the organization, evolution and diversity of soybean miRNA families. For example, in the three miRNA families that play a crucial role in auxin signaling (miR160, miR164 and miR393), only one family member had been identified previously (miRBase V17). Our results show that these three miRNA families have 6, 12 and 11 members respectively in soybean (Additional file 1: Table S1). Such knowledge is crucial for complete understanding of redundancy and diversity between miRNA family members in the regulation of plant growth and development. With high levels of conservation in mature sequences, what is the need for larger families of miRNAs? Is there diversification of function among family members or does one member play a major role and functional redundancy exists among family members? There is evidence for both possibilities. In Arabidopsis, the three family members of miR164 have overlapping expression domains and individual loss of function mutants in each of these miRNAs have distinct phenotypes whereas the triple mutant has additive phenotypes [50,51]. On the other hand, miR159a and b single mutants do not have obvious developmental phenotypes but the double mutant exhibits severe developmental defects, suggesting functional redundancy among family members [7]. In addition, we have identified five novel (previously undescribed) miRNAs and all of them seem to be soybean-specific although reads with significant identity to gma-new-miR13587 were also present in a *M. truncatula* sRNA library (MIRMED; [26]; Subramanian, unpublished results). It is noteworthy that gma-new-miR11602 was independently identified in soybean by Kulcheski et al. [24] and reads corresponding to mature sequences of all 5 novel miRNAs identified in our study were present in multiple soybean small RNA libraries (NCBI GEO archive) suggesting that these are indeed genuine miRNAs. Three of them (gma-new-miR21193, gma-new-miR13587, gma-new-miR50841) had high abundance in root or nodule libraries (data not shown) consistent with our qPCR data on gma-new-miR13587 and gma-new-miR50841.

### Genome organization of soybean miRNAs

**Figure 6:**
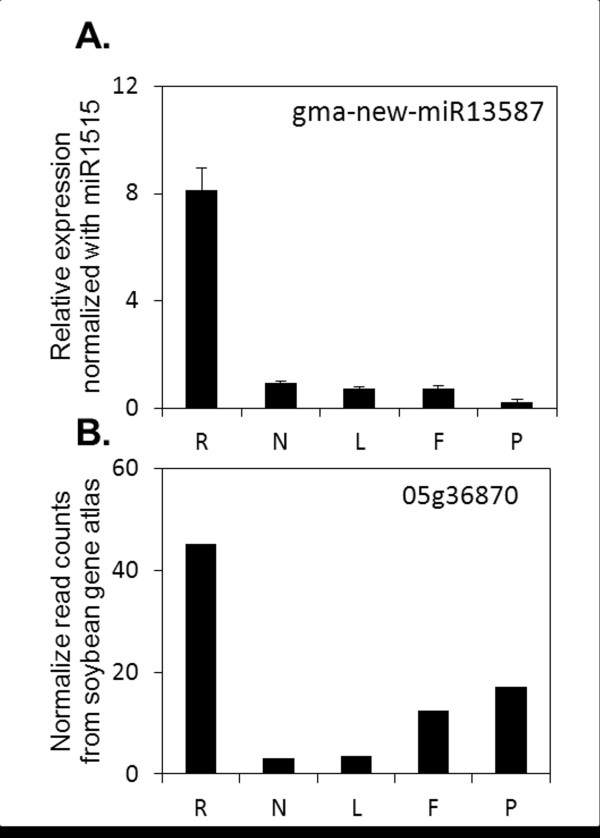
**Expression of gma-new-miR13587 and its ‘parent gene’ (Glyma05g36870) in different soybean organs. A. **Expression of gma-new-miR13587 was assayed by RT-qPCR and normalized to miR1515. **B. **Expression of Glyma05g36870 is presented as normalized read counts from soybean gene atlas. Data were obtained from (http://digbio.missouri.edu/soybean_atlas/ (Libault *et al.*[49]). Root (R), Nodule (N), Leaf (L), Flower (F) and Pod (P).

Soybean miRNA genes are primarily intergenic as in other plant species [4,7,26]. We also identified several soybean miRNAs that were either intragenic or very closely located to a protein-coding gene (Additional file 3: Table S2). In animals, intronic miRNAs are much more frequently observed than for plants and a co-regulation between the miRNA and its parent gene is often suggested [52-54]. In plants, it has been hypothesized that the presence of non-coding RNAs in introns could have implications for the biogenesis of both mature small RNAs and host mRNA [55]. In particular, a potential competition between host gene splicing and miRNA production could occur and it is possible that splicing events are influenced by the pre-assembly of the pri-miRNA processing complexes (e.g. ath-MIR838 and DCL1; [10]). So far, no evidence has been found in plants for co-regulation of miRNA and the parent gene. We compared the organ level expression of gma-new-miR13587 to its ‘parent gene’, Glyma05g36870 (obtained from soybean gene atlas; [49]). Both of them had a strong preferential expression in roots (Figure 6), but were poorly expressed in other tissues, suggesting potential co-regulation of gene expression. Comparing the expression of other miRNA-parent gene pairs in soybean might reveal novel insights into potential co-regulation of signaling pathways by these partners.

We identified four miRNA families that have tandemly duplicated members: MIR159, MIR169, MIR395, three conserved families, and MIR-Seq14 that is soybean-specific. In addition to these tandem duplications, we also observed four miRNA clusters (groups of miRNAs located within 5Kb of each other with no protein coding genes in between) involving members from different families (Additional file 4: Table S3). miRNA clustering is often conserved among distantly related angiosperms [20]. Clusters of miRNAs in plant genomes generally contain conserved miRNAs of the same family, in contrast to animals where miRNAs with unrelated sequences are often included in the same clusters [56]. In all families with tandemly repeated miRNAs in soybean, the number and/or orientation of miRNA and miRNA-like genes in paralogous genomic loci was different (Figure 2 and Additional file 2: Figure S2), suggesting that miRNA evolution and diversification was not only due to whole genome duplication events, but also independent local duplication of miRNA genes. However, even though duplication/clustering of miRNA genes can be found in distantly related angiosperms, it does not seem to be a generally conserved phenomenon. For example, like in soybean, clusters of MIR159 genes have been identified in maize and sorghum as well [57], whereas miR159 family is encoded by three unclustered genes in Arabidopsis [58]. Similarly, MIR395 is organized in clusters in different plant species such as Arabidopsis and rice but not in *M. truncatula*[26,59]. MIR169 is also organized in cluster in several species including rice [60] and Arabidopsis [61] and its organization highly conserved with *G. max*[20].

### Continuous evolution and diversification of soybean miRNAs

miRNA genes are evolving rapidly; evolutionary dynamics also influences miRNA family size, diversity of mature sequences among family members, length of mature sequence and the number of targets regulated [57]. The notion that many plant miRNA families are conserved but others are lineage specific has been clearly supported by comparisons of miRNA inventories, especially between two closely related Brassicaceae plants, *A.thaliana* and *A. lyrata*[16,42,43]. miRNA genes that are deeply conserved among plant lineage tend to belong to families that have several members, possess quite conserved mature sequences that are shorter (primarily 21nt) and usually regulate a conserved set of genes; In contrast, the less-conserved miRNAs are a much larger category, usually represented by a few members with higher mature sequence diversity, longer mature miRNAs (primarily 22nt) and often with fewer or no targets. It is hypothesized that some less conserved miRNA families may be nonfunctional and evolutionarily transient [7,16,42,43,62]. In soybean as well, conserved, legume-specific and soybean-specific gene families had distinct characteristics that were in agreement with the above observations in Arabidopsis.

### Generation of secondary siRNAs by legume miRNA families

A number of legume-specific miRNAs members seemed to target disease resistance genes. It is consistent with the fact that disease resistant genes are often highly species- or plant-family-specific. In addition, miR2109 and miR1510, both legume-specific miRNAs co-target five different disease-resistance genes. We also found other pairs of miRNAs that appear to co-target several genes and each pair tended to target genes encoding proteins of similar function (e.g. miR1508 and miR4413 targeting genes encoding PPR-repeat containing proteins). This suggests an extra layer of regulation that might finely tune expression of these genes. For example, these miRNAs might regulate the targets in different tissues, in response to different pathogens or could act to generate tasiRNAs from these loci [48]. Indeed we observed a higher than median production of phased siRNAs from genes encoding three PPR repeat-containing proteins. Consistent with our results, a recent publication also reported two of these genes as phased siRNA-producing loci [47]. Interesting hypotheses on the significance of tasi-RNA generation form R-genes include formation of a gradient to delineate functional boundaries, organ/cell type-specific silencing of gene families and even a possible role in unequal meiotic crossover frequencies often observed in specific gene families.

### Potential regulation of nodule-specific gene expression by miRNAs

We also identified a set of soybean miRNAs and corresponding targets that had inverse expression pattern between nodule and roots, and are therefore potentially involved in the regulation of the nodulation process. In *M. truncatula,* restriction of MtHAP2-1 expression to the meristem by miR169a was shown to be crucial for proper nodule development in *M. truncatula*[31]. Despite the absence of a meristem in soybean nodules, two targets of miR169, Glyma10g10240 and Glyma17g05920, which encode HAP proteins were highly expressed in the nodules (Figure 5E-F). More interestingly, the expression of miR169 genes was very low in nodules. Similarly, gma-new-miR13587 was highly expressed in the roots, but poorly in nodules and three of its potential targets presented an inverse expression pattern (Figure 5A-C). These observations suggested that nodule-specific expression of genes could be regulated by the presence or absence of miRNAs in a particular organ in soybean.

## Conclusions

In conclusion, our study examined the organization of miRNA families in soybean genome and identified possible evolutionary mechanisms associated with functional diversification. The study also provides a comprehensive overview of miRNA diversity and family characteristics in soybean. Consistent with recent reports in Arabidopsis, family size, diversity, length and nucleotide distribution of mature sequences, and the range of targets regulated by them were distinct between conserved and legume- or soybean-specific miRNA families. Finally, inverse expression patterns of specific miRNA-target pairs in different tissues (e.g. roots vs nodules) suggested regulation of organ-specific gene expression by miRNAs in soybean.

## Material and methods

Plant growth and *B. japonicum* treatment (adapted from [18]).

Soybean (*Glycine max* cv. Williams82) seeds were surface-sterilized with 10% clorox for 4 minutes followed by 70% ethanol for 2 minutes. They were then rinsed 6 times with sterile deionized water and planted in 4” pots (about 15 seeds per pot) filled with 1:2 (v/v) mixture of sterilized perlite and vermiculite (Hummert International, St Louis, MO). Pots were watered regularly with ¼ x nitrogen free plant nutrient solution (N- PNS) and maintained in a controlled environment plant growth chamber (16 h light; 25°C; 30% relative humidity). *B. japonicum* cells (USDA110) were grown and 12 days old seedlings inoculated as described in Subramanian *et al.*[18].

Bioinformatics analyses:

1. miRNA identificationSmall RNA isolation, library construction and sequencing are described in Subramanian *et al.*[18]. Bioinformatics assisted analyses were essentially same as Subramanian *et al.*[18] except that prediction of precursor sequences was performed using complete chromosome assembly of soybean (Glyma1; http://www.phytozome.net;[36). Reads with perfect match to the genome in more than 35 positions were also discarded from further analysis. In Arabidopsis, it has been suggested that reads with matches to more than 20 positions in the genome are more likely to be repeat sequences or transposons rather than miRNAs [10]. Compared to an estimated 1.15 fold duplication in Arabidopsis [63], soybean genome has a 2.55 fold duplication. To account for this higher duplication in soybean, we increased this number to 35. [64,65]. In addition, potential miRNAs were selected based on criteria for annotation of plant miRNAs described by Meyers *et al*. [17]. The primary criterion is evidence for precise excision of the miRNA/miRNA* duplex with a two nucleotides overhang in 3’ end. The most abundant strand, therefore considered as the miRNA, should have no more than 4 mismatches with its corresponding miRNA* (especially within the duplex), including a maximum of 1 asymmetrical bulge of 1 or 2 nucleotides. The duplex should represent a minimum of ~25% observed reads from the stem-loop region of the precursor. In the absence of miRNA*, a significant abundance of the miRNA strand is required (we used a threshold of 10 reads in our library of ~350,000 reads). Presence of the potential miRNA sequence in multiple/independent smallRNA libraries also strengthens the validation. Secondary criteria were defined to strengthen plant miRNA annotation, but are not sufficient by themselves. These include conservation in sequence with established miRNAs with a maximum of 3 nucleotides mismatch with the potential miRNA and prediction of potential target genes in the genome. Potential precursor sequences confirming to the above criteria were annotated as miRNAs. Representative sequences were assigned to each miRNA locus based on read abundance and uniqueness of the mature sequence for each locus. The most represented mature sequence was generally selected as representative ID by default. Exceptions were made if this particular sequence was included in another mature sequence, meaning exact same sequence except for additional nucleotides in 3’ and/or 5’ end, and had a read count corresponded to at least 10% of the total abundance in a particular locus. In that case, the longer mature sequence was selected as representative ID1 and the shorter sequence as representative ID2.

2. List of soybean miRNAsA list of all miRNAs identified in soybean was established, including miRNAs identified in this study, those already available in miRbase, valid miRNAs listed in Zhang *et al*.[20] and plant miRNA data base [38] and the publicly available sequences from articles published during the preparation of this manuscript [22-24]. We examined all of these miRNAs if they fit the criteria established by the community for annotation of plant miRNAs [17]. Only those miRNAs that passed the criteria were included in subsequent analyses. Similarly, all miRNA loci/genes predicted to produce 24nt mature miRNA species were also excluded from further analysis due to their ambiguity being long-miRNAs (lmiRNAs) or heterochromatic-siRNAs (hc-siRNAs)[39].

3. Genome organizationThe position of miRNA genes in the soybean genome (Glyma109) was identified using BLAST searches. Diagrammatic representation of genome organization of miRNAs was obtained using circos software [66]. The relative position of miRNA genes with respect to protein-coding genes was identified using a custom perlscript (available upon request). Illustrations of genome elements were obtained using the soybean genome browser at soybase.org. Phylogenetic trees were constructed using MEGA4 [67] after CLUSTALW alignment of miRNA precursor sequences. The evolutionary history was inferred using the Neighbor-Joining method [68] and the optimal tree is shown (Figure 2 and Additional file 2: Figure S2). The evolutionary distances were computed using the Maximum Composite Likelihood method [69] and are in the units of the number of base substitutions per site. Positions containing gaps and missing data in all sequences were eliminated from the dataset (Complete deletion option).

4. Family characteristics:The miRNAs were clustered into families using miRBase criteria: 0 to 2 mismatches being typical, but up to 4 was considered acceptable. One adjustment was made compared to the suggested family ID given by miRBase: based on mature sequence similarities with members of both miR2118 and miR2218 families, members of these families were designated as belonging to the miR2118/2218 family. For each miRNA family, the number of family members, number and size of each unique mature sequence were analyzed. A 2 tail heteroscedastic (Two-sample unequal variance) Student’s T-test was used to analyze the difference between conserved, legume and soybean-specific families for those characteristics and number of target predicted.

### Target prediction and validation

The criteria used for target identification were: maximum 1 mismatch between the 2^nd^ and 9^th^ nt, no mismatch at the 10^th^ and 11^th^ nts, maximum 4 mismatches after the 12^th^ nt with no more than 2 consecutive mismatches and a calculated minimum free energy of at least 70% of a perfect complement between miRNA and its target (referred as target prediction score) [8,9].

Targets were validated using a modified 5’RLM-RACE (RNA ligase-mediated rapid amplification of 5’ cDNA ends) assay. First, polyA mRNA was isolated from total RNA using PolyAtract mRNA isolation systems (Promega Z5300). 5’RLM-RACE was performed on the resulting mRNA preparation using Generacer Kit (Invitrogen, Carlsbad, CA, product No.450168) omitting calf intestinal phosphatase and tobacco acid pyrophosphatase steps to allow ligation of RNA adapter to cleaved transcripts. Nested PCR was performed (Primer sequences for PCR and nested-PCR in Additional file 4: Table S5) following cDNA synthesis and PCR products were gel purified using “Wizard SV gel and PCR clean up system” (Promega, Madison, WI, product No. A9682) and then cloned using “TOPO TA Cloning kit for sequencing” (Invitrogen, Carlsbad, CA, product No. K4575) and sequenced (Beckman Coulter Genomics, Boston, MA).

### Tissue harvest and RNA isolation

For expression analysis in different soybean organs, samples were collected from: root, stem and young leaves of 12 days old plants, 14dpi mature nodules, flowers and young pods. A biological replicate that included the tissues of minimum 10 plants and three technical replicates were performed. All tissues were immediately frozen in liquid N_2_ and stored at −80°C. All root tissues harvested were first washed thoroughly and blot dried.

RNA extraction was performed using Trizol (Invitrogen, Carlsbad, CA). Approximately 1 g of tissue powder was mixed with 10 ml of Trizol and centrifuged (5000xg, 10 min) to remove debris. The supernatant was transferred to a new tube, mixed well with 2 ml of chloroform and centrifuged (5000xg, 10 min). Chloroform extraction was repeated twice with the aqueous phase containing RNA. The resulting supernatant was precipitated overnight at −20°C with an equal volume of isopropanol. After centrifugation (12000xg,10 min), the RNA pellet was washed with 10 ml of 70% ethanol and resuspended in 50 μl of DEPC-treated water. RNA quality was estimated using a Nanodrop ND-1000 spectrophotometer (Nanodrop, Wilmington, DE) and by running an aliquot on an agarose gel.

### Gene expression analysis by RT qPCR for miRNAs and target genes

cDNA synthesis for miRNA gene expression was performed with 2 μg RNA, using hairpin primers specific to each mature miRNA sequence essentially as described by Varkonyi *et al.*[70] (Additional file 3: Table S5) using M-MuLV reverse transcriptase (NEB, Ipswich, MA) and miR1515 as reference gene [33].

cDNA synthesis for target genes was performed using oligo-dT and M-MuLV reverse transcriptase (NEB, Ipswich, MA [71]). 2 μg RNA were mixed with 1 μl of 10 μM oligodT and dNTP mix (10 mM each) to a final volume of 16.5 μl. The mixture was incubated at 75°C for 5 minutes followed by 5 minutes on ice. Subsequently, 0.5 μl of the enzyme with corresponding buffer and 0.25 μl of RNase out (Invitrogen, Carlsbad, CA) were added and cDNA synthesis carried out at 42°C for 1 h. Finally, the enzyme was inactivated by incubating 95°C for 5 minutes.

qPCR assays were performed using a Stratagene MX3000P equipment (Stratagene, La Jolla, CA) and SYBR premix (Clontech, Mountain View, CA). For miRNA gene expression, one cycle of 95°C for 10 seconds, 45 cycles of the following: 95°C for 5 seconds , 60°C for 10 seconds and 72°C for 1 second were performed and followed by standard dissociation curve assay (95°C for 1 minute, 55°C for 30 seconds and 95°C for 30 seconds). For target gene expression, one cycle of 95°C for 10 seconds, 40 cycles of the following: 95°C for 5 seconds and 64°C for 20 seconds were performed and followed by standard dissociation curve assay (95°C for 1 minute, 55°C for 30 seconds and 95°C for 30 seconds). List of primers used for qPCR are presented in (Additional file 3: Table S5) and dissociation curves and linearity of amplification in (Additional file 2: Figure S4). We considered a variation in expression level when a difference of at least two fold was observed.

## Competing interests

The authors declare that they have no competing interests.

## Authors’ contributions

SS conceived and designed the study. SS and OY coordinated the project. SS and MT did the experiments, bioinformatics analysis and wrote the manuscript. All authors read and approved the final manuscript.

## Supplementary Material

Additional file 1 **Table S1. **List of miRNA genes identified and used in this study. Click here for file

Additional file 2 **Figure S1. **Novel miRNAs identified in this study. Figure S2. Tandem duplication of MIR159 and MIR395 genes. Figure S3. Nucleotide distribution of mature miRNA sequences from different classes of soybean miRNA families. Figure S4. Dissociation curves and amplification efficiency of qPCR assays. Click here for file

Additional file 3 **Table S2. **Intragenic soybean miRNAs, and miRNAs located less than 1Kb from a protein-coding gene. **Table S4. **miRNA targets tested negative for miRNA-mediated cleavage as assayed by a modified 5’RLM-RACE assay. **Table S5. **List of primers used in this study. Click here for file

Additional file 4 **Table S3. **Target prediction for all available miRNAs in soybean. Click here for file
