# Supplementary material for: Genome organization and characteristics of soybean microRNAs
Source: BMC Genomics. 2012 May 4;13:169. doi: 10.1186/1471-2164-13-169 (PMC3481472; doi:10.1186/1471-2164-13-169)
Supplement: Additional file 2 — Figure S1. Novel miRNAs identified in this study. Figure S2. Tandem duplication of MIR159 and MIR395 genes. Figure S3. Nucleotide distribution of mature miRNA sequences from different classes of soybean miRNA families. Figure S4. Dissociation curves and amplification efficiency of qPCR assays. [file 1471-2164-13-169-S2.pdf]

Table of contents:

Figure S1	P2
Figure S2	P3-5
Figure S3	P6
Figure S4	P7-8

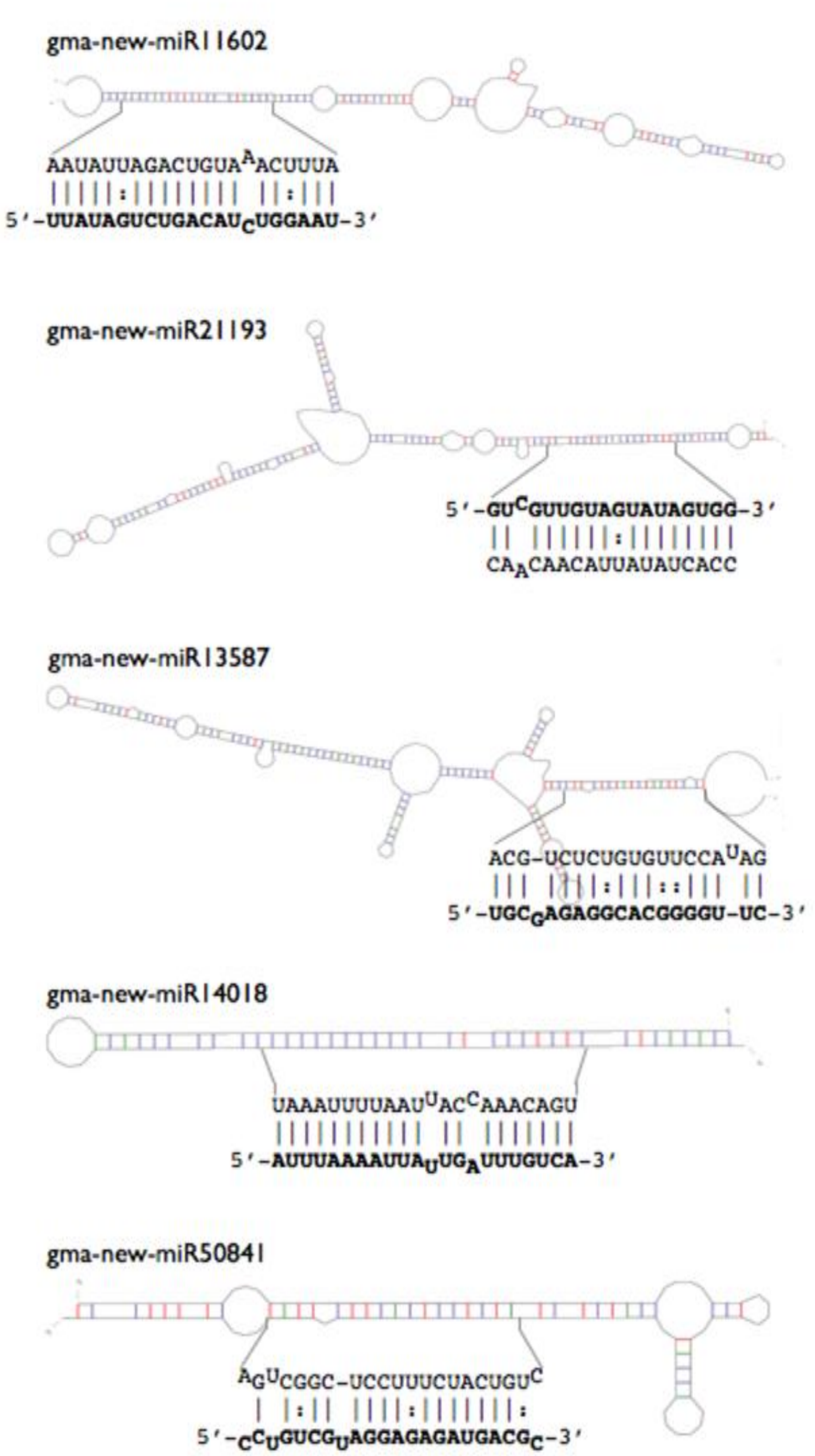

Figure S1. Novel miRNAs identified in this study. Minimum free energy folding structures of precursors are shown with miRNA-miRNA* duplexes enlarged. miRNA sequences are shown in bold face.

A.

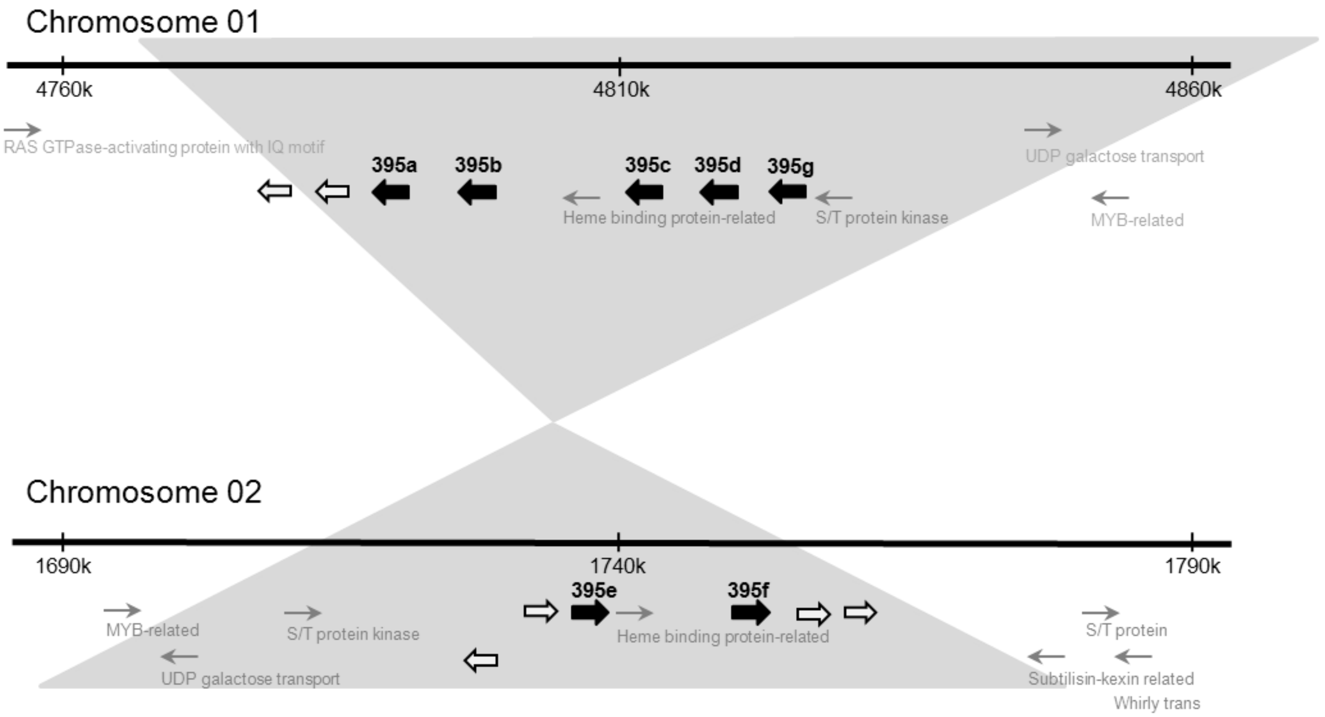

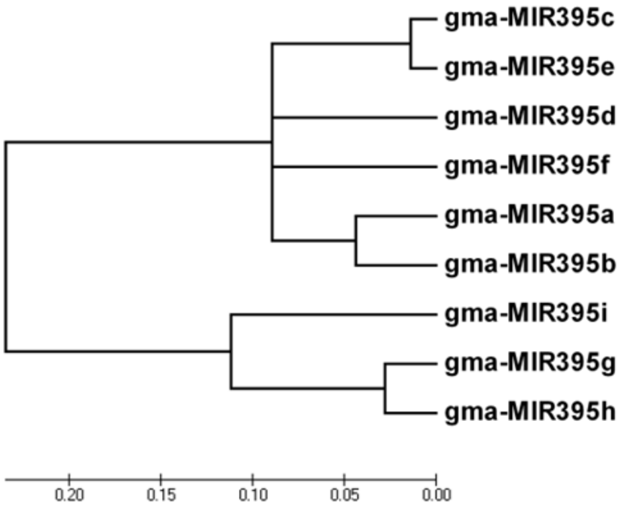

miRNA family member	Mature sequence
gma-MIR395a	AUGAAGUGUUUGGAGAACUC
gma-MIR395b-f	AUGAAGUGUUUGGGGAACUC
gma-MIR395g-i	CUGAAGUGUUUGGGGAACUC

B.

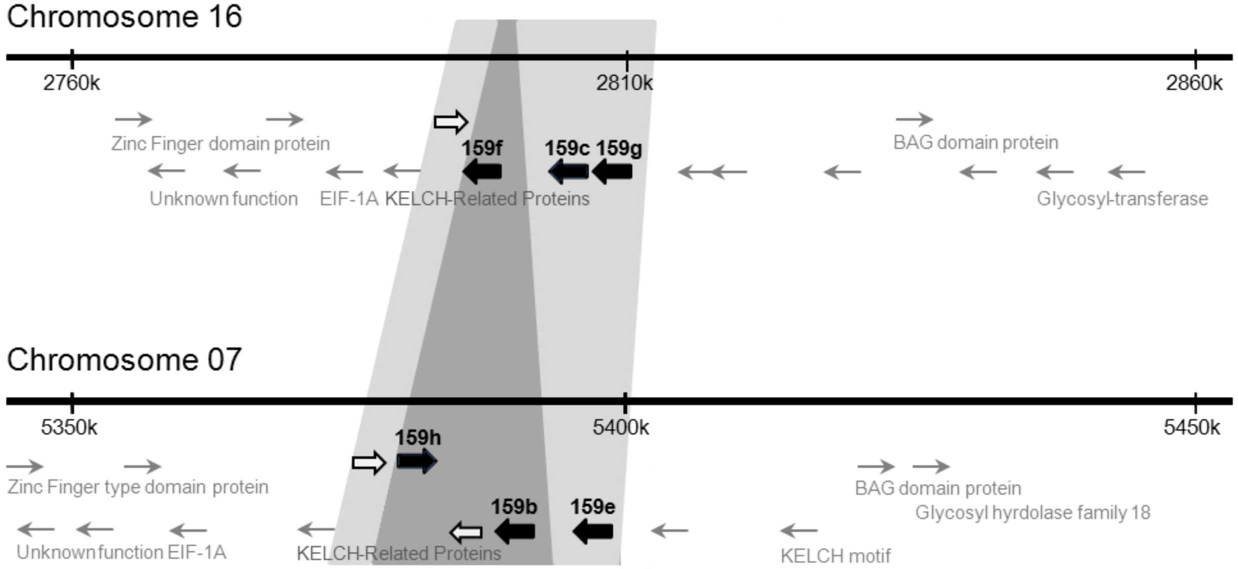

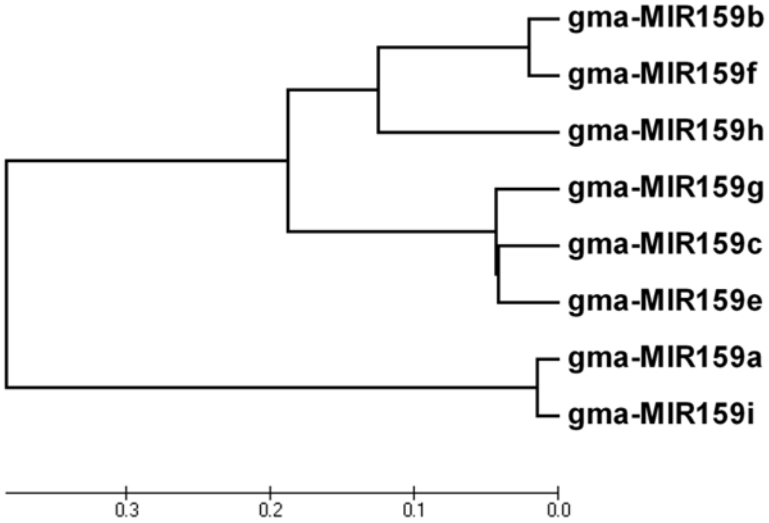

miRNA family member	Mature sequence
gma-MIR159a,i3p	UUUGGAUUGAAGGGAGCUCUA
gma-MIR159b	AUUGGAGUGAAGGGAGCUCCA
gma-MIR159c	AUUGGAGUGAAGGGAGCUCCG
gma-MIR159d	AGCUGCUUAGCUAUGGAUCCC
gma-MIR159e	AUUGGAGUGAAGGGAGCUCCU
gma-MIR159f,g	AUUGGAGUGAAGGGAGCUC
gma-MIR159h	AUUUGAGUGAAGGGAGCUCCG

Figure S2:

A. Illustration showing a portion of soybean chromosome 16 with tandemly duplicated MIR159 genes (filled arrows), MIR159-like genes (hollow arrows) and its duplicated region on chromosome 7. While both paralogous genomic loci have tandemly duplicated miRNA genes, the orientation of genes is not the same (Notice gma-MIR159h on the opposite strand compared to other genes). This observation suggested that at least part of the miRNA duplication occurred more recently compared to genome duplication. Examination of the phylogenetic tree obtained by aligning the precursor sequences of all soybean MIR159 genes suggests that MIR159e, c and g form a closely related group while MIR159b, f and h form another related group. miRNAs and miRNA-like genes with high sequence identity are shown encompassed in background boxes.
B. Illustration showing a portion of soybean chromosome 1 with tandemly duplicated MIR395 genes (filled arrows), MIR395-like genes (hollow arrows) and its duplicated region on chromosome 2 (paralogous genomic loci are indicated by grey background). Presence of tandemly duplicated miRNA genes on only one of the multiplicons suggests that miRNA duplication occurred more recently compared to genome duplication. miRNAs and miRNA-like genes with high sequence identity are shown encompassed in background boxes.

Position of miRNA genes were identified through BLAST searches. Illustrations of genome elements were obtained using the soybean genome browser at soybase.org. A custom annotation track (can be downloaded from our lab website: http://www.sdstate.edu/ps/faculty/senthilsubramania/) was used to mark the positions of miRNA genes in the soybean genome. Grey arrows indicate the position of protein-coding genes. Annotations specified where available (Arrows not to scale of gene length).

Phylogenetic trees were constructed using MEGA4 after CLUSTALW alignment of miRNA precursor sequences. The evolutionary history was inferred using the Neighbor-Joining method Saitou *et al*. (1987). The optimal tree with the sum of branch length = 0.51092826 is shown. The evolutionary distances were computed using the Maximum Composite Likelihood method Tamura *et al.* (2004) and are in the units of the number of base substitutions per site. All positions containing gaps and missing data were eliminated from the dataset (Complete deletion option). There were a total of 95 positions in the final dataset. Phylogenetic analyses were conducted in MEGA4 Tamura *et al.* (2007).

Literature cited:

Saitou N, Nei M (1987) The neighbor-joining method: a new method for reconstructing phylogenetic trees. Mol Biol Evol **4:** 406-425

Tamura K, Dudley J, Nei M, Kumar S (2007) MEGA4: Molecular Evolutionary Genetics Analysis (MEGA) software version 4.0. Mol Biol Evol **24:** 1596-1599

Tamura K, Nei M, Kumar S (2004) Prospects for inferring very large phylogenies by using the neighbor-joining method. Proc Natl Acad Sci U S A **101:** 11030-11035

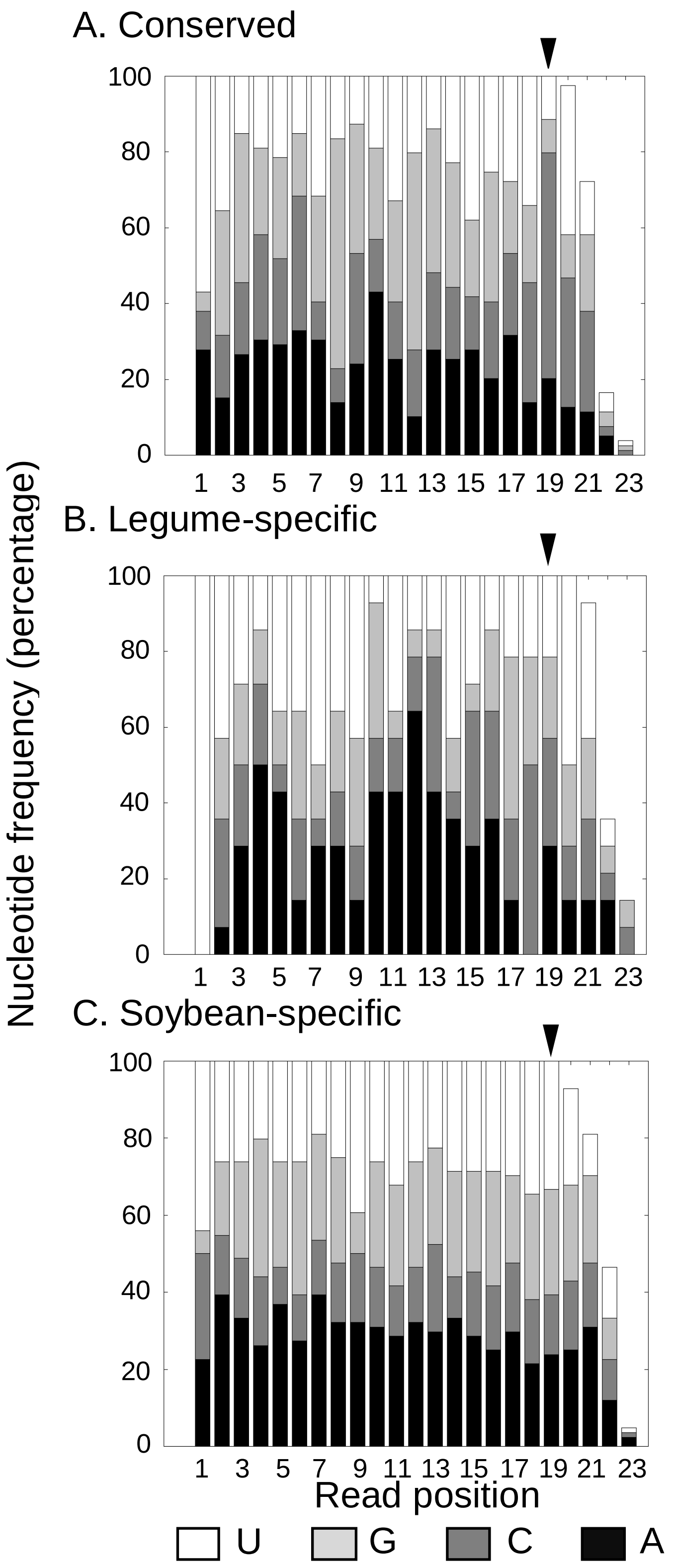

Figure S3. Nucleotide distribution (U (white), G (light grey), C (dark grey), A (black)) of mature miRNA sequences from different classes of soybean miRNA families. **A**. conserved (79 sequences) **B**. Legume-specific (14 sequences) and **C**. Soybean-specific (85 sequences miRNAs) had distinct nucleotide preference at 19th positions (indicated with arrow heads). Nucleotide distribution analysis was performed using Fastx tool kit (http://hannonlab.cshl.edu/fastx_toolkit/). Potential correlation of these criteria with the level of conservation of the family was examined. Since the data is in suppl data, methods can go there as part of figure legend.

A.

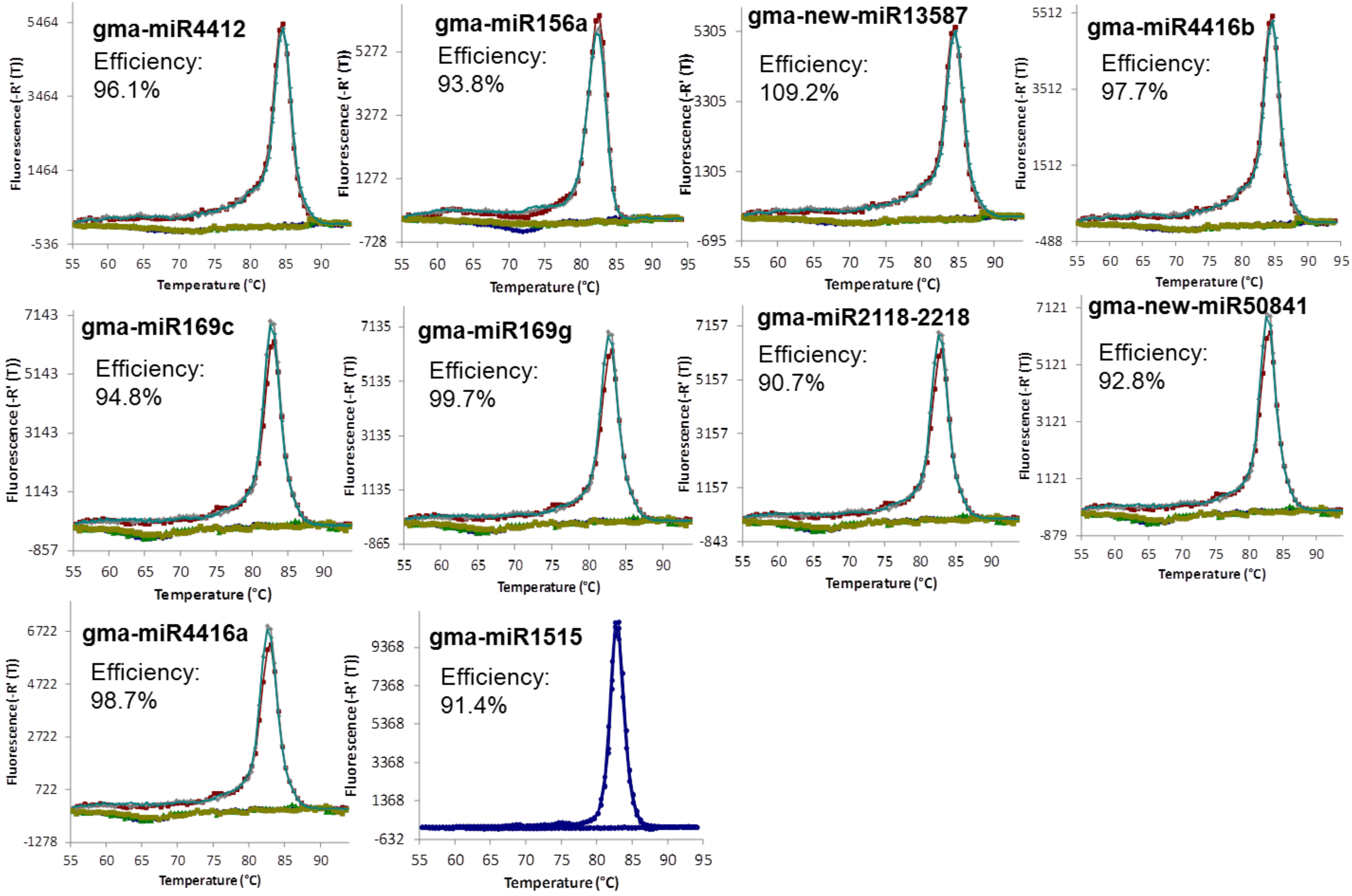
gma-miR4412
Efficiency: 96.1%
gma-miR156a
Efficiency: 93.8%
gma-new-miR13587
Efficiency: 109.2%
gma-miR4416b
Efficiency: 97.7%
gma-miR169c
Efficiency: 94.8%
gma-miR169g
Efficiency: 99.7%
gma-miR2118-2218
Efficiency: 90.7%
gma-new-miR50841
Efficiency: 92.8%
gma-miR4416a
Efficiency: 98.7%
gma-miR1515
Efficiency: 91.4%
Fluorescence (-R' (T))
Temperature (°C)

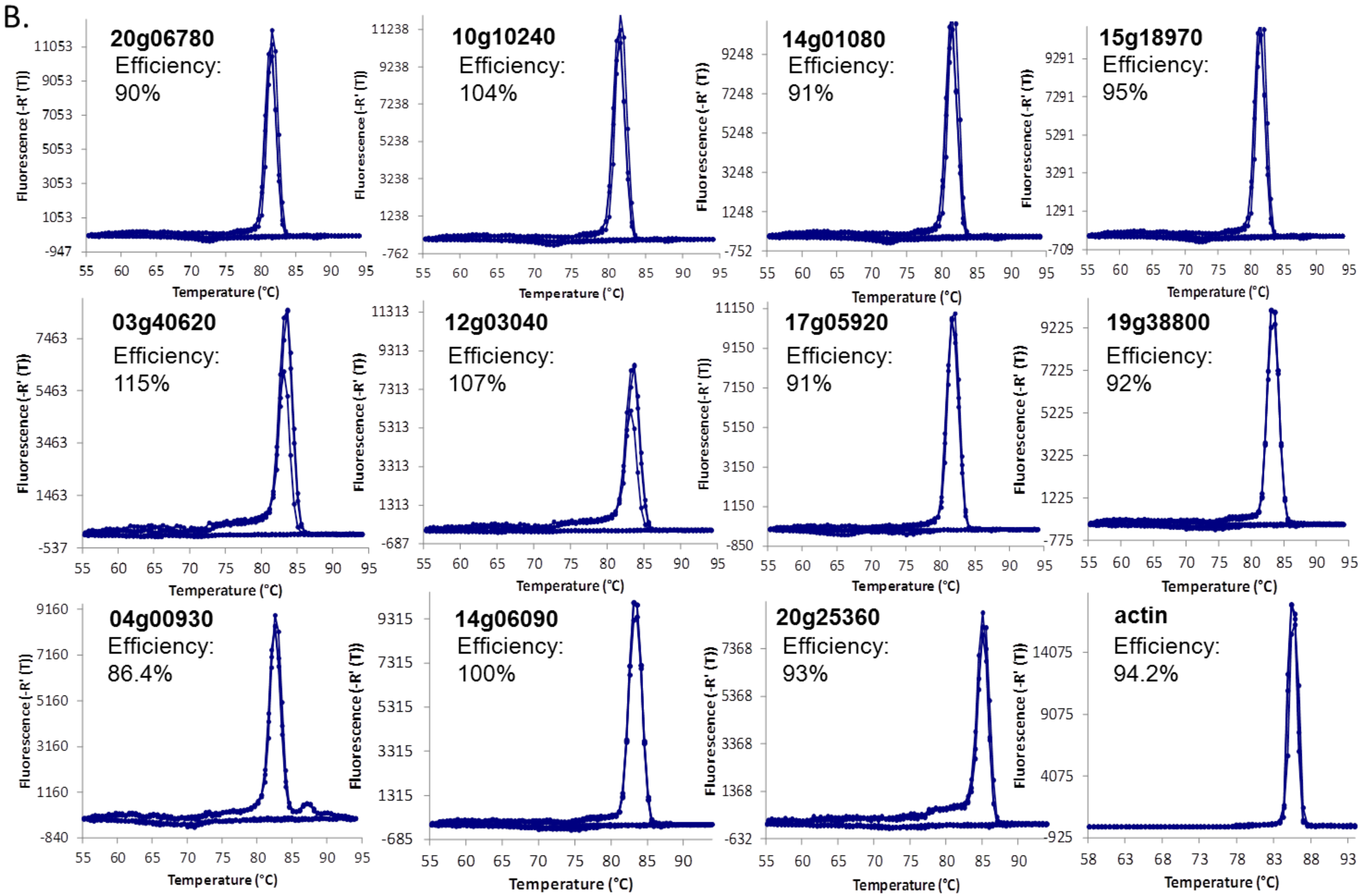

Figure S4: Dissociation curves and amplification efficiency of qPCR reactions to assay **A**. miRNA and **B.** target genes. For all miRNA and target genes assayed, we tested our primers using root cDNA non diluted, 1/10, 1/100 and 1/1000 times diluted. We selected for further analysis only the genes for which 1. the primer pair presented a nice single peak dissociation curve, 2. no non-specific amplification (primer-dimers) and 3. a linear amplification (i.e. Amplification efficiency 100% + or- 10%).
