# Supplementary material for: Genome organization and characteristics of soybean microRNAs
Source: BMC Genomics. 2012 May 4;13:169. doi: 10.1186/1471-2164-13-169 (PMC3481472; doi:10.1186/1471-2164-13-169)
Supplement: Additional file 3 — Table S2. Intragenic soybean miRNAs, and miRNAs located less than 1Kb from a protein-coding gene. Table S4. miRNA targets tested negative for miRNA-mediated cleavage as assayed by a modified 5’RLM-RACE assay. Table S5. List of primers used in this study. [file 1471-2164-13-169-S3.pdf]

Table of content:

Table S1:	see Additional Table S1.xls
Table S2:	P.2-3
Table S3:	see Additional Table S3.xls
Table S4:	P.4-5
Table S5:	P.6-8

Table S2. Intragenic soybean miRNAs (A) and miRNAs located less than 1Kb from a protein-coding gene (B) and their relative position compared to the parent gene (protein coding genes containing miRNA genes (A)) or proximal gene (protein coding genes located less than 1Kb from miRNA genes (B)).

A.

miRNA	Parent gene	Predicted function of parent gene	miRNA location
gma-MIR4340	Glyma19g02330	Protein kinase	intron CDS
gma-MIR4351	Glyma19g38070	F-Box domain protein	3'UTR[1]
gma-MIR-Seq10	Glyma15g28840	Serine/threonine protein kinase	intron CDS[1]
gma-MIR-Seq12	Glyma16g23790	LRR-containing protein	exon CDS[1]
gma-MIR-Seq17	Glyma16g03450.	Uncharacterized protein	intron CDS[1]
soy_9	Glyma05g10140	Phosphoglycerate mutase family	intron CDS
soy_18	Glyma10g12660	Hydrolase	intron CDS[1]

1. Gene prediction with FgeneSH identified additional genes compared to phytozome annotation.

B.

miRNA	Proximal gene	miRNA position	Distance from proximal gene	Proximal gene predicted function
gma-MIR1520a	Glyma14g28370	5'	972	Glycosyl transferase
gma-MIR4387e	Glyma03g19830	3’	933	No functional annotation
gma-MIR4397	Glyma04g31190	5'	678	No functional annotation
gma-MIR4399	Glyma03g19830	3'	458	No functional annotation
gma-new-MIR21193	Glyma08g04500	5'	238	Peptidyl-tRNA hydrolase
gma-new-MIR13587	Glyma05g36870	3'	748	Ring finger protein
soy_23	Glyma02g16190	5’	668	No functional annotation

The relative position of miRNA genes with respect to protein-coding genes was identified using a custom perlscript. For proximal genes of miRNAs, gene structure prediction was independently verified using FgeneSH using Medicago as reference species.
(http://linux1.softberry.com/berry.phtml?topic=fgenesh&group=programs&subgroup=gfind).

Table S4: miRNA targets not validated by a modified 5'RACE assay. Inability to detect cleavage by 5'-RACE could also be due to miRNA regulation in specific tissue/cell-types within the roots. Additional experiments (e.g. tissue-specific expression of miRNA and target pairs) are necessary to examine such a possibility.

<table>
<tr><th>miRNA</th><th>Target name and function</th><th>Target prediction score [1]</th><th>5'RACE result[2]</th><th></th></tr>
<tr><td>gma-miR156a</td><td>Glyma03g40620
MAPKK-RELATED</td><td>0.83</td><td>3'CACGAGTGAGAGAAGACAGT 5'
||||| |||||||||||
ATGCTCTCTCTCTTCTGTCT</td><td>No amplification</td></tr>
<tr><td>gma-miR169c</td><td>Glyma14g01080
PENTATRICOPEPTIDE REPEAT-CONTAINING PROTEIN</td><td>0.8</td><td>3'AGCCGTTCAGTAGGAACCGAA 5'
| ||| ||| || |||||||
TGGGCGAGTTATTCTTGGCTA</td><td>0/15</td></tr>
<tr><td rowspan="8">gma-new-miR13587</td><td>Glyma02g08270
ubiquitin thiolesterase</td><td>0.71</td><td>3'CTTGGGGCACGGAGAGCGT 5'
| ||| |||||||||
AATCCCGCTGCCTCTCGTT</td><td>0/11</td></tr>
<tr><td>Glyma04g00930
amidotransferases</td><td>0.78</td><td>3'TGGGGCACGGAGAGCGT 5'
||| |||||||||||
CCCCTCTGCCTCTCGCA</td><td>0/15</td></tr>
<tr><td>Glyma06g07570
monooxygenase</td><td>0.71</td><td>3'TGGGGCACGGAGAGCGT 5'
|| |||||||||| ||
GCCGCGTGCCTCTCCCA</td><td>No amplification</td></tr>
<tr><td>Glyma14g06090
DNA repair protein, HELICASE</td><td>0.81</td><td>3'TGGGGCACGGAGAGCGT 5'
||||||||| ||||
TGCCCGTGCCTTTCGCC</td><td>0/11</td></tr>
<tr><td>Glyma17g23850
none</td><td>0.83</td><td>3'TGGGGCACGGAGAGCGT 5'
|| |||||||||||
GGCCTGTGCCTCTCGCT</td><td>No amplification</td></tr>
<tr><td>Glyma19g38290
F-box family protein</td><td>0.72</td><td>3'CTTGGGGCACGGAGAGCGT 5'
| | ||||||||||||
GCTCTGCGTGCCTCTCGCG</td><td>0/6</td></tr>
<tr><td>Glyma20g08730
KELCH-RELATED PROTEINS</td><td>0.8</td><td>3'TGGGGCACGGAGAGCGT 5'
|| ||||| ||||||
CCCACGTGCTTCTCGCG</td><td>0/7</td></tr>
<tr><td>Glyma20g25360
PROTEIN PHOSPHATASE 2C</td><td>0.73</td><td>3'TGGGGCACGGAGAGCGT 5'
| | |||||||| ||
ATCTTGTGCCTCTTGCT</td><td>0/10</td></tr>
</table>

miRNA	Target name and function	Target prediction score [1]	5'RACE result[2]	
**gma-new-miR21193**	Glyma01g07640 SIK1; ATP binding / protein kinase	0.85	3'**TGGTGATATGATGTTGCTG** 5'<br>|| || ||||| ||||| GCCGCTGGACTACGACGAC	0/8
	Glyma02g13220 SIK1; ATP binding / protein kinase	0.85	3'**TGGTGATATGATGTTGCTG** 5'<br>|| || ||||| ||||| GCCGCTGGACTACGACGAC	0/10
	Glyma08g26150 SEC14 cytosolic factor family protein	0.7	3'**TGGTGATATGATGTTGCTG** 5'<br>|| | |||||||||| CCCGGTGTACTACAACGTG	No amplification
	Glyma11g37820 glycosyl transferase family 17 protein	0.71	3'**TGGTGATATGATGTTGCTG** 5'<br>|||| ||||||||||| TCCACATTACTACAACGAA	0/9
	Glyma12g00390 SEC14 cytosolic factor family protein	0.7	3'**TGGTGATATGATGTTGCTG** 5'<br>|| | ||||||||||| | CCCGGTGTACTACAACGTC	0/11
	Glyma17g34200 unknown protein	0.72	3'**TGGTGATATGATGTTGCTG** 5'<br>||||| | |||||| ||| ACCACAAGACTACAGCGAT	0/9
	Glyma18g01740 glycosyl transferase family 17 protein	0.71	3'**TGGTGATATGATGTTGCTG** 5'<br>|||| ||||||||||| TCCACATTACTACAACGAA	0/10

1. Ratio of minimum free energy compared to perfect complementary pairing between miRNA and target
2. Base-pairing between miRNA and its target are shown. Numbers indicate number of clones indicating cleavage at the predicted cleavage site and the number of clones sequenced.
3. miRNA is shown in bold face.

Table S5. List of primers for **A.** cDNA synthesis, **B.** miRNA qPCR, **C.** targets qPCR and **D.** 5'RACE.

A. miRNA specific cDNA synthesis primers

Primer sequence 5'-3'	miRNA
GTCGTATCCAGTGCAGGGTCCGAGGTATTCGCACTGGATACGACcagatc	gma-miR1515
GTCGTATCCAGTGCAGGGTCCGAGGTATTCGCACTGGATACGACgtgctc	gma-MIR156a
GTCGTATCCAGTGCAGGGTCCGAGGTATTCGCACTGGATACGACatattg	gma-MIR171g
GTCGTATCCAGTGCAGGGTCCGAGGTATTCGCACTGGATACGACgaagga	gma-MIR319c
GTCGTATCCAGTGCAGGGTCCGAGGTATTCGCACTGGATACGACtcggca	gma-MIR169c
GTCGTATCCAGTGCAGGGTCCGAGGTATTCGCACTGGATACGACggcaag	gma-MIR169g
GTCGTATCCAGTGCAGGGTCCGAGGTATTCGCACTGGATACGACtaggaa	gma-MIR2118/2218
GTCGTATCCAGTGCAGGGTCCGAGGTATTCGCACTGGATACGACctccag	gma-MIR4416b
GTCGTATCCAGTGCAGGGTCCGAGGTATTCGCACTGGATACGACaccccg	gma-new-miR13587
GTCGTATCCAGTGCAGGGTCCGAGGTATTCGCACTGGATACGACgcgtca	gma-new-miR50841
GTCGTATCCAGTGCAGGGTCCGAGGTATTCGCACTGGATACGACgaggca	gma-MIR4412
GTCGTATCCAGTGCAGGGTCCGAGGTATTCGCACTGGATACGACcctagg	gma-MIR4416a

B. qPCR primers for miRNA

Primer sequence 5'-3'	Primer ID (universal reverse and miRNA specific forward)
GTGCAGGGTCCGAGGT	universal reverse (Varkonyi *et al*. 2007)
TCGCTtcattttgcgtgcaat	gma-miR1515-F
TCGCTtgacagaagagagt	gma-MIR156a-F
TCGCTtgattgagccgtgc	gma-MIR171g-F
TCGCTtggactgaagggagc	gma-MIR319c-F
TCGCTaagccaaggatgact	gma-MIR169c-F
TCGCTcagccaaggatga	gma-MIR169g-F
TCGCTttgccgattccaccca	gma-MIR2118/2218-F
TCGCTacgggtcgctctcac	gma-MIR4416b-F
TCGCTtgcgagaggca	gma-new-miR13587-F
TCGCTcctgtcgtaggagaga	gma-new-miR50841-F
TCGCTtgttgcgggtatctt	gma-MIR4412-F
TCGCTtacgggtcgctctca	gma-MIR4416a-F

Varkonyi-Gasic E, Wu R, Wood M, Walton EF, Hellens RP (2007) Protocol: a highly sensitive RT-PCR method for detection and quantification of microRNAs. Plant Methods **3:** 12

C. qPCR primers for targets

miRNA	Primer sequence 5’-3’	Targets
gma-MIR156a	TTCTGGGTCATTTCGAGGTC	03g40620.1F
	GAAGATGATGGTGCCCAAGT	03g40620.1R
gma-MIR2118/2218	CACTAATTGTCTGCTAGGGATTCAT	12g03040.1F
	CTAAAGTTGGAGAGAAAACACGAAC	12g03040.1R
	TCGGACGAGAGTATCGAGTT	20g06780.1F
	CGTCAAACTGCTTGTAAATGGA	20g06780.1R
gma-MIR169c and gma-MIR169g	AGGGACCAGGTGAGGAACTT	10g10240.1F
	TGACAAGCCTGTGCAGAAAG	10g10240.1R
	GGTCAGGCTGGCAACTTAAA	14g01080.1F
	TTGAACAGCCTCAGTCATGC	14g01080.1R
	GCAACGCACTTCAGTTGTCC	15g18970.1F
	CCAAAGCAATTGGAAAGGTC	15g18970.1R
	CGATGAGAGGTGGAACCAAT	17g05920.1F
	TGGGAAAGCAGTTCCAAAAG	17g05920.1R
	GCGTGTGTTGGCTGTGATAA	19g38800.1F
	ACAGGTACATCGAGCCAAGG	19g38800.1R
gma-new-miR13587	CAAATCCCTTCCAAACCAAA	04g00930.1F
	CGTCGTCGAAGAAGACACAA	04g00930.1R
	GTTCATGGCCCTATCATCGT	06g07570.1F
	ACGTAGGGTACTCCGTGGTG	06g07570.1R
	TGCTTGTGCTGAAGGCATTA	14g06090.1F
	TCCAATGAACCTGTCACCAA	14g06090.1R
	TGCCCTTCTATCTTCGGAAA	17g23850.1F
	GAAAGGGCACGACATGAGAT	17g23850.1R
	CCACGGAACAGAATTTACCG	20g08730.1F
	CCGCATGGCTAGTGAGTACA	20g08730.1R
	TTTGGTGGGGCTAGATATGG	20g25360.1F
	CCCTTTCTTGGACTGAGCTG	20g25360.1R

D. 5'RACE primers

miRNA	Primer sequence 5'-3'	Targets
gma-MIR156a	CAATTAGGAAGAGATTTTCCTCTCC	03g40620.1R nested
	TATGCTTCACAAGAAGAGCAGAACC	03g40620.1R
gma- MIR2118/2218	CTAAAGTTGGAGAGAAAACACGAACC	12g03040.1R nested
	GTATCTCTGATAGATGCCCTTCTTG	12g03040.1R
	CTGTCTTAGGATTTGAGGTTTCTCC	20g06780.1R nested
	TTTGTCGTCCTCAAGTATCTCTGAT	20g06780.1R
gma-MIR169c and gma-MIR169g	ACACATCATAGTCTACTGCATCAGG	10g10240.1R nested
	GAAATCCAGAACAAAATACCACACC	10g10240.1R
	ATCAACTTAGTGCCCAAGCTACTTT	14g01080.1R nested
	CAGGATCACCATGAGTAGAATTGTT	14g01080.1R
	AGTGCTAATGCAGACTGAAGAGTCT	15g18970.1R nested
	ACTAGTTCCATGCACAGACTATTCC	15g18970.1R
	CCAAAAGACAACATTTCTGTAGCAC	17g05920.1R nested
	TTCAAGCACAAACTTTCTGGTCCAG	17g05920.1R
	ACAGGTACATCGAGCCAAGGATGAG	19g38800.1R nested
	GTCTTCTGCATCAGGATATCAAGTC	19g38800.1R
gma-new-miR13587	CGTCGAAGAAGACACAAGTGTTTTG	04g00930.1R nested
	GCCAGGGAGCAGAGACGGGAGGCTT	04g00930.1R
	GTGTTTAGGGAGGTGGAGTTTGAG	06g07570.1R nested
	AGCGTAGGAGTTGGAGGGGTATTT	06g07570.1R
	CTCTTTCCAAGTGGTTCTTTTGTAC	14g06090.1R nested
	ATCATCTTCAATCTTCTCAGCTTGCC	14g06090.1R
	CACCTCATTCTCATCATATGGAAAGG	17g23850.1R nested
	TATTTCTTCCGCAGAAGGAAACTCG	17g23850.1R
	GCGCCTCCACGTCGAACAAATCCAT	20g08730.1R nested
	CCGCATGGCTAGTGAGTACATACAC	20g08730.1R
	TCCAACCCTCACAGTAGGCTTCTCC	20g25360.1R nested
	ATGATGAAGAGTTCCCAGGCACTCG	20g25360.1R
